# Patients With Obsessive-Compulsive Disorder Exhibit Deficits in Consummatory but Not Anticipatory Pleasure

**DOI:** 10.3389/fpsyg.2019.01196

**Published:** 2019-05-24

**Authors:** Sihui Li, Yi Zhang, Jie Fan, Wanting Liu, Jun Gan, Jing He, Jinyao Yi, Changliang Tan, Xiongzhao Zhu

**Affiliations:** ^1^Medical Psychological Center, The Second Xiangya Hospital, Central South University, Changsha, China; ^2^Medical Psychological Institute of Central South University, Changsha, China; ^3^Department of Radiology, The Second Xiangya Hospital, Central South University, Changsha, China

**Keywords:** obsessive-compulsive disorder, depression, anhedonia, consummatory, anticipatory

## Abstract

**Background:** Reward dysfunctions have been reported in obsessive-compulsive disorder (OCD), which implicates a high possibility of anhedonia for this disease. However, several components of anhedonia, such as consummatory and anticipatory pleasure, has not been substantially studied in OCD patients.

**Methods:** The Chinese version of the Temporal Experience of Pleasure Scale (CV-TEPS) was used to evaluate both the consummatory and anticipatory pleasure in 130 OCD patients, 89 major depressive disorder (MDD) patients, and 95 healthy controls (HCs). The Yale-Brown Obsessive-Compulsive Scale (Y-BOCS) and the Beck Depression Inventory (BDI) were scored for assessing the severity of obsessive and compulsive symptoms and depressive symptoms, respectively. Analyses of covariance (ANCOVA) were used to compare the differences of anhedonia among the three groups with the severity of depression controlled. Regression analyses were also used to analyze the relationship between consummatory and anticipatory pleasure and clinical variables in OCD patients.

**Results:** After controlling for the effect of depression, there were significant differences in TEPS scores among the three groups (*p* < 0.05). Compared with HCs, OCD patients had lower scores on the consummatory subscale, but not the anticipatory subscale, of the TEPS. MDD patients had lower scores on both the consummatory and anticipatory subscales than HCs.

**Conclusion:** OCD patients exhibit deficits in consummatory but not anticipatory pleasure, which is distinct from MDD patients.

## Introduction

Anhedonia is an impaired and diminished capacity to experience pleasure (Clark and Fawcett, 1987). It is considered as one of the core symptoms of major depressive disorder (MDD), and moreover, as one of the potential pharmacological targets (Giannantonio et al., 2011; Martinotti et al., 2012; De Berardis et al., 2016; Giovanni et al., 2016). Anhedonia was also observed in other psychiatric disorders (Isella et al., 2003; Hasler et al., 2004; Strauss et al., 2011). For example, a recent study has revealed that 28.3% of the patients with obsessive-compulsive disorder (OCD) demonstrated anhedonia symptoms (Abramovitch et al., 2014), suggesting the possibility that anhedonia is also a feature of OCD. However, to date, very limited studies have investigated anhedonia in OCD patients.

One way to parse pleasure is to divide it into different components, which might be associated with different reward processes (Berridge and Robinson, 2003). Previous studies have explored the physical anhedonia and found the absence of sexual pleasure in OCD patients (Vulink et al., 2006; Çeşmeci et al., 2017). Some researchers suggest that anticipatory pleasure, a feeling of wanting which is related to goal-directed motivation and expected reward, should be distinguished from consummatory pleasure, which is concentrated on “instant” or real-time experience when answering to pleasurable stimulation, and related to a sense of satiation or a resolution of desire (Klein, 1984; Berridge and Robinson, 1998). To our knowledge, no studies to date have investigated the characteristics of anticipatory and consummatory pleasure in OCD patients.

When repeating thoughts and behaviors, OCD patients usually had negative emotions, such as anxiety and depression, which might lead to seldom pleasure experienced by patients at that moment (Ferreira et al., 2017). On the other hand, OCD patients expected to attain anticipatory reward from their compulsive behaviors (Leckman et al., 1994; Fontenelle et al., 2015). Driven by perfectionistic tendencies, they always believed that the results of the future were better than now (Foa and Kozak, 1986). Thus, it can be proposed that OCD patients mainly exhibit deficits in the consummatory pleasure but can still expect for the anticipatory pleasure.

It should be noted that most patients with OCD have an increased depression level (Fineberg et al., 2005) and the association between depression and anhedonia is generally accepted (Pizzagalli et al., 2009). Chasson et al. (2017) suggested that anhedonia might be one comorbidity mechanism shared by obsessive-compulsive and depressive symptoms in adolescents. Thus, it is important to control the effect of depression when investigating anhedonia in patients with OCD. To do this, in the present study, we have controlled the depression level to clarify that anhedonia in OCD patients was not caused by their increased depression level. Moreover, unlike what we hypothesized for OCD, most previous studies demonstrated that MDD patients were impaired in both anticipatory and consummatory pleasure (Li et al., 2015; Zhang et al., 2016). Hence, we also compared anhedonia characteristics between OCD and MDD to illustrate differences in pleasure deficits between these two mental disorders.

The Temporal Experience of Pleasure Scale (TEPS) was developed to especially assess the anticipatory and consummatory pleasure (Gard et al., 2006). It consists of two subscales: anticipatory subscale and consummatory subscale. Chan et al. (2012) revised a Chinese version of the TEPS (CV-TEPS) and demonstrated that for the CV-TEPS, both the consummatory and anticipatory subscales could be further divided into abstract and contextual factors. The contextual factor involved concrete scenarios, while the abstract factor involved board and less concrete scenarios. However, nearly all researchers still adopted the 2-factor structure in their studies (Chan et al., 2010; Li et al., 2015). Hence, in the present study, we also used the anticipatory subscale (TEPS-ANT) and consummatory subscale of the TEPS (TEPS-CONS) to investigate the pleasure deficits in OCD patients.

To sum up, the current study aimed to explore the characteristics of consummatory and anticipatory pleasure in OCD patients, by comparing the consummatory and anticipatory anhedonia among OCD patients, MDD patients and the healthy controls (HCs). Our hypothesis was that OCD patients mainly exhibited deficits in consummatory but not anticipatory pleasure.

## Materials and Methods

### Participants

A total of 142 patients with OCD and 96 patients with MDD were screened consecutively from the psychological clinic of the Second Xiangya Hospital of Central South University between December 2015 and February 2018. Patients with any comorbid axis I psychiatric disorders and a history of severe organic or neurologic pathology, such as head injury, were excluded from this study. Diagnosis was established using structured clinical interview for axis I disorders (SCID-I) of the Diagnostic Statistical Manual of Mental Disorders, Fourth Edition (DSM-IV) by two psychiatrists. After screening, three OCD patients and 2 MDD patients withdrew consents. Nine OCD patients and 5 MDD patients were excluded because of the comorbidity with other psychiatric disorders. The remained 130 OCD patients and 89 MDD patients enrolled in the study. Among them, 57 OCD patients and 26 MDD patients were under pharmacological treatment (see details in Table 1).

**TABLE 1:** Comparisons of demographic and clinical characteristics among OCD patients, MDD patients and healthy controls HCs.

	**OCD (*n* = 130)**	**MDD (*n* = 89)**	**HCs (*n* = 95)**
Age (years)	23.58 ± 8.60	26.17 ± 10.37	25.07 ± 6.50
Gender (male/female)	71/59	40/49	42/53
Education (years)	12.99 ± 3.29	13.92 ± 2.35	15.02 ± 2.89
General IQ	117.02 ± 8.72	116.45 ± 7.40	119.21 ± 8.85
BDI	18.19 ± 11.13	28.96 ± 10.43	4.67 ± 5.95
Medication, yes/no	57/73	26/63	–
Medication type			
SSRIs	53	18	–
SNRIs	3	5	–
NaSSA	1	3	–
Duration of illness (year)	3.88 ± 4.80	1.88 ± 2.22	–
Y-BOCS	19.96 ± 5.95	–	–
Y-BOCS-O	11.16 ± 3.16	–	–
Y-BOCS-C	8.80 ± 4.26	–	–
Y-BOCS-insight	1.72 ± 1.02		

*MDD, major depressive disorder; OCD, obsessive-compulsive disorder; HCs, healthy controls; IQ, intelligence quotient; BDI, Beck Depression Inventory; Y-BOCS, The Yale-Brown Obsessive Compulsive Scale; Y-BOCS-C, compulsive-subscale of Y-BOCS; Y-BOCS-O, obsessive-subscale of Y-BOCS; Y-BOCS-insight, item 11 of the Y-BOCS; SSRIs, selective serotonin reuptake inhibitors; SNRIs, serotonin-norepinephrine reuptake inhibitors; NaSSA, noradrenergic and specific serotonergic antidepressant.*

The HCs were recruited from colleges and communities and evaluated using the structured clinical interview for disorders-non-patient edition (SCID-NP). Participants with a history of psychiatric disorders and severe organic or neurologic pathology were excluded. Ninety-five participants were screened and none of them was excluded.

This study was approved by the Ethics Committee of the Second Xiangya Hospital, Central South University. All subjects were voluntarily involved and signed the informed consent.

### Measures

The Chinese version of the Temporal Experience of Pleasure Scale (CV-TEPS) was used to evaluate the different components of pleasure (Chan et al., 2012). It consists of 20 items with 11 items for anticipatory pleasure and 9 items for consummatory pleasure. Each item was rated on a 6-point Likert scale. The CV-TEPS showed good reliability and validity in patients with MDD (Li et al., 2015). In this study, the CV-TEPS showed good internal consistency (Cronbach’s α = 0.84) and test-retest reliability (*r* = 0.63) in OCD patients.

The Yale-Brown Obsessive-Compulsive Scale (Y-BOCS) was used to measure the severity of obsessive and compulsive symptoms and the insight level of the patients (Goodman et al., 1989). It is composed of 11 items with 5 items for assessing the severity of obsessive thoughts and 5 for assessing the severity of compulsive behaviors. Item 11 of the Y-BOCS was used to assess the insight level. Each item was scored from 0 to 4. OCD patients with a total score of 6–15 were classified as mild, 16–25 as moderate, and above 25 as severe. The Y-BOCS showed good internal consistency in OCD patients in this study (Cronbach’s α = 0.79).

The Beck Depression Inventory (BDI) was adopted to assess the depression level (Beck et al., 1961). It comprised 21 items with each item being scored from 0 to 3 by the Likert four-point scale. Participants with a score of less than or equal to 9 were classified as minimal depression, 10 to 18 as mild depression, 19 to 29 as moderate depression, and over or equal to 30 scores as severe depression. The Chinese version of the BDI had good reliability and validity to measure depression level (Wang et al., 2011). In this study, the BDI showed good internal consistency in OCD patients (Cronbach’s α = 0.90).

General intelligence of each subject was evaluated with the Chinese version of the Wechsler Abbreviated Scale of Intelligence (Gong, 1982). The CV-TEPS and the BDI were administrated to all the participants, while the Y-BOCS was only finished by the OCD patients.

### Data Analysis

Data were analyzed using SPSS version 21 (IBM, United States). One-way analyses of variance (ANOVA) or chi-square tests were used to compare the demographic variables among OCD group, MDD group and HCs. Analyses of covariance (ANCOVA) were used to compare the differences of anhedonia among the three groups after controlling the severity of depression. Regression analyses were used to analyze the relationship between anhedonia and clinical variables in patients with OCD. See Supplementary Materials for additional analysis of effect of insight on anhedonia in OCD.

## Results

### Demographic and Clinical Characteristics of Participants

As shown in Table 1, there were no significant differences in age (*F*_2,311_ = 2.49, *p* > 0.05) and gender (χ^2^ = 3.09, *p* > 0.05) among OCD, MDD, and HCs. Each subject had a general intelligence score greater than 90 and showed similar general intelligence across groups (*F*_2,311_ = 2.87, *p* > 0.05). In addition, there was no significant difference in medication status (χ^2^ = 4.81, *p* > 0.05) between OCD and MDD patients. However, both patient groups had lower education levels than HCs (*F*_2,311_ = 13.26, *p* < 0.05; *post hoc* OCD < HCs, MDD < HCs, all *p* < 0.05). The BDI scores of OCD and MDD groups were significantly higher than HCs (*F*_2,311_ = 147.12, *p* < 0.05; *post hoc* MDD > OCD > HCs, all *p* < 0.05). The OCD patients had significantly longer duration of illness than MDD patients (*t* = 3.66, *p* < 0.01).

### Comparison of Anhedonic Characteristics Among the Three Groups

The BDI scores and education levels were controlled as covariates when comparing the differences of TEPS scores among the three groups. ANCOVA showed significant group effects on anticipatory pleasure (*F*_2,311_ = 9.49, *p* < 0.01, η^2^ = 0.06), and consummatory pleasure (*F*_2,311_ = 24.66, *p* < 0.01, η^2^ = 0.14). Specifically, using Bonferroni correction, *post hoc* tests revealed a trend of MDD < OCD < HCs in consummatory pleasure and MDD < HCs in anticipatory pleasure (all *p* < 0.01). There was no significant difference in anticipatory pleasure between OCD and HCs (Table 2). There were no significant gender differences in TEPS scores in OCD patients (Supplementary Table S1).

**TABLE 2:** Comparisons of consummatory and anticipatory pleasure among OCD patients, MDD patients, and healthy controls HCs.

	**OCD**	**MDD**	**HCs**	***F***	***p***
TEPS-ANT	33.03 ± 6.88	27.65 ± 8.67	34.57 ± 6.10	9.49	<0.01
TEPS-CONS	37.25 ± 8.92	31.06 ± 6.62	41.92 ± 7.43	24.66	<0.01

*MDD, major depressive disorder; OCD, obsessive-compulsive disorder; HCs, healthy controls; TEPS, Temporal Experience of Pleasure Scale; TEPS-ANT, anticipatory subscale of the TEPS; TEPS-CONS, consummatory subscale of the TEPS. Anticipatory scores: MDD < HCs (*p* < 0.01), MDD < OCD (*p* < 0.01); consummatory scores: MDD < OCD < HCs (all *p* < 0.01); All results in *post hoc* tests were adjusted by Bonferroni correction.*

### Regression Analysis

Regression analyses were performed on scores of TEPS-ANT and TEPS-CONS, respectively. In the both models, Y-BOCS-C scores, Y-BOCS-O scores, the level of insight, BDI scores, duration of illness, and the medication treatment status were entered as independent variables. No significant associations were observed for both models [all *p* ≥ 0.040, not significant with Bonferroni correction (0.05/6); Tables 3, 4].

**TABLE 3:** Predictors of anticipatory pleasure in patient with OCD.

	***B***	***SD B***	**β**	***t***	**Partial *r***	***P***
Y-BOCS-C	-0.041	0.152	-0.025	-0.267	-0.024	0.790
Y-BOCS-O	0.171	0.225	0.078	0.757	0.068	0.450
Y-BOCS_ insight	-0.166	0.657	-0.025	-0.252	-0.023	0.802
BDI	-0.015	0.059	-0.024	-0.254	-0.023	0.800
Duration of illness	0.078	0.129	-0.055	-0.605	-0.054	0.546
Medication treatment	-0.204	1.262	-0.015	-0.162	-0.015	0.872

*OCD, obsessive-compulsive disorder; BDI, Beck Depression Inventory; Y-BOCS, The Yale-Brown Obsessive-Compulsive Scale; Y-BOCS-C, compulsive-subscale of Y-BOCS; Y-BOCS-O, obsessive-subscale of Y-BOCS; Y-BOCS-insight, item 11 of the Y-BOCS. Model statistics, *R^2^* = 0.008, Corrected *R^2^* = −0.041, *F*_6,123_ = 0.16, *p* = 0.987.*

**TABLE 4:** Predictors of consummatory pleasure in patients with OCD.

	***B***	***SD B***	**β**	***t***	**Partial *r***	***p***
Y-BOCS-C	-0.397	0.191	-0.189	-2.074	-0.184	0.040
Y-BOCS-O	0.108	0.283	0.038	0.381	0.034	0.704
The score of insight	-0.272	0.825	-0.031	-0.329	-0.030	0.742
BDI	0.086	0.074	0.107	1.161	0.104	0.248
Duration of illness	0.129	0.162	0.070	0.795	0.072	0.428
Medication treatment	-2.614	1.584	-0.146	-1.650	-0.147	0.101

*OCD, obsessive-compulsive disorder; BDI, Beck Depression Inventory; Y-BOCS, The Yale-Brown Obsessive-Compulsive Scale; Y-BOCS-C, compulsive-subscale of Y-BOCS; Y-BOCS-O, obsessive-subscale of Y-BOCS; Y-BOCS-insight, item 11 of the Y-BOCS. Model statistics, *R^2^* = 0.072, Corrected *R*^2^ = 0.027, *F*_6,123_ = 1.60, *p* = 0.153.*

## Discussion

This study demonstrated that, with the severity of depression controlled, OCD patients were impaired in consummatory pleasure but not anticipatory pleasure. In contrast, MDD patients were impaired in both the consummatory and anticipatory pleasure. Several strengths of the current study should be noted. First, to the best of our knowledge, it was the first study in which the characteristics of deficits in anticipatory and consummatory pleasure had been revealed in OCD patients. Second, we have investigated anhedonia in OCD patients with the effect of depression removed. Third, the differences of anhedonia between OCD and MDD patients were revealed. These findings indicated that deficit in consummatory pleasure in OCD was not caused by the severity of depression, which might deepen our understanding in the psychopathological mechanism of OCD. In addition, by revealing the anhedonia differences in OCD and MDD, our results can not only enrich the theoretical research of anhedonia but also contribute to the identification of these two mental disorders.

Consistent with the most current findings (Sherdell et al., 2012; Li et al., 2015), we found that MDD patients suffered both the consummatory and anticipatory anhedonia, although there are also few previous studies observed deficits only in the anticipatory pleasure in MDD patients (McFarland and Klein, 2009). Previous studies suggested that MDD patients lost interest in daily life blunted not only in emotional reactivity to positive stimuli, but also to anticipated reward (Bylsma et al., 2008; McFarland and Klein, 2009), which was evidenced by our results. In contrast, the characteristics of anhedonia in OCD were rarely reported, and no study has focused on the consummatory and anticipatory pleasure in OCD patients. In this study, we first explored both components in OCD patients, and found that consummatory pleasure was selectively impaired in OCD patients. In previous anhedonia-related studies in OCD (Abramovitch et al., 2014; Chasson et al., 2017), researchers treated anhedonia as a unitary construct and paid less attention to the different aspects of anhedonia, which might cause inconsistent results.

In this study, the characteristics of consummatory and anticipatory pleasure in patients with OCD were distinct from those in patients with MDD, with OCD patients exhibiting no deficits in anticipatory pleasure. Previous studies have suggested some neurobiological distinctions in reward processing between patients with OCD and MDD (Remijnse et al., 2009; Figee et al., 2010). The dysfunction of the reward system, included emotion and motivation pathways, plays an important role in the neural basis of anhedonia (Gard et al., 2006). While patients with MDD were impaired in both the emotional and motivational pathways (Zhang et al., 2013; Lu and Yuan, 2014), OCD patients mainly exhibited dysfunctional brain circuits underlying emotional processing which corresponds to consummatory pleasure (Figee et al., 2010; Lu and Yuan, 2014). Thus, we can propose that the distinctions in pleasure deficits between OCD and MDD may be caused by different deficits in the rewarding process. Further research is required to determine whether these neurobiological distinctions in reward processing could account for the different abnormal performance of anhedonia between OCD and MDD.

Abramovitch et al. (2014) reported that about 1/3 OCD patients experienced clinically significant anhedonia even after controlling for depressive symptoms. This study further found that impairments in consummatory pleasure in OCD could not be attribute to depression symptoms. The regression analysis in the present study also revealed that anhedonia in OCD was not correlated with other clinical variables, which suggested that anhedonia might be simply another symptom of OCD. Ferreira et al. (2017) study found that compulsive washing may be more clearly characterized by problems in reward processing; in contrast, duration of checking, severity of OCD, and comorbidity with impulse control disorders shaped compulsive behaviors by imparting them with habitual tendencies. These findings implied that anhedonia may be related to some specific symptoms rather than all types or total severity of compulsion or obsession (Figee et al., 2010). In addition, Treadway and Zald (2011) found that the first-line antidepressants such as SSRIs, SNRIs, and NaSSA were not satisfying in the treatment of anhedonia, which could possibly explain why medication treatment had no significant effect on anhedonia in OCD in this study.

Some limitations in this study should be addressed. First, the current study adopted convenient sampling to recruit patients with OCD from clinic, and fifty-seven OCD patients were on antidepressant medications. Although this study found no differences in the characteristics of anhedonia between medicated and unmedicated OCD patients (Supplementary Table S2), the medication effect should be further controlled in future study. A medication-free OCD group will be more warranted. Second, this cross-sectional study just used questionnaire method and did not conduct behavior paradigms, such as reward tasks relating to anhedonia. It is significant to combine multiple methods to perform longitudinal studies on different reward phases in patients with OCD. Third, pleasure can not only be treated as a unitary concept, but also be parsed into different aspects. For example, based on the different phases of reward process, there are consummatory and anticipatory components of pleasure. According to the nature of the pleasurable stimulus, pleasure can be classified as social and physical aspects. Moreover, pleasure can also be classified as state and enduring personality trait characteristics. Correspondingly, different self-report measures, such as the Snaith – Hamilton Pleasure Scale (SHAPS; Snaith et al., 1995), the TEPS, the Revised Physical Anhedonia Scale (RPAS; Chapman et al., 1976), the Social Anhedonia Scale (RSAS; Chapman et al., 1976) and the Anticipatory and Consummatory Interpersonal Pleasure Scale (ACIPS; Gooding and Pflum, 2014), have been developed to assess pleasure. Basing our hypothesis, we only used the TEPS in this study, which focused on measuring the consummatory and anticipatory components of pleasure. Thus, our finding, combined with previous findings of deficits in physical and sexual pleasure in OCD (Vulink et al., 2006; Çeşmeci et al., 2017), cannot reveal the whole picture of pleasure deficits in OCD patients. Future studies are necessary to address this problem by systematically adopting those different measures. Last, although the current study indicated that the characteristics of pleasure deficits in patients with OCD were distinct from those in patients with MDD, more intensive studies to explore the cause of the discrepancy are required.

## Conclusion

This study found that OCD patients exhibit deficits in consummatory but not anticipatory pleasure, which is distinct from MDD patients.

## Ethics Statement

This study was carried out in accordance with the recommendations of the Ethics Committee of the Second Xiangya Hospital, Central South University with written informed consent from all subjects. All subjects gave written informed consent in accordance with the Declaration of Helsinki. The protocol was approved by the Ethics Committee of the Second Xiangya Hospital, Central South University.

## Author Contributions

SL conceived and designed the study. XZ had full access to all the data in the study and took responsibility for the integrity of the data and the accuracy of the data analysis. JG, WL, and JH extracted the data. SL and JF analyzed the data. SL, YZ, and XZ contributed to interpret the data. JY and CT revised the manuscript. SL wrote the first draft of the manuscript. All authors critically revised the manuscript and approved the final version.

## Conflict of Interest Statement

The authors declare that the research was conducted in the absence of any commercial or financial relationships that could be construed as a potential conflict of interest.
