# Supplementary material for: Patients With Obsessive-Compulsive Disorder Exhibit Deficits in Consummatory but Not Anticipatory Pleasure
Source: Front Psychol. 2019 May 24;10:1196. doi: 10.3389/fpsyg.2019.01196 (PMC6558405; doi:10.3389/fpsyg.2019.01196)
Supplement: Supplementary file 1 [file Table_1.DOCX]

SUPPLEMENTARY MATERIALS

**Patients with obsessive-compulsive disorder exhibit deficits in consummatory but not anticipatory pleasure**

***Results***

**Table S1.** Comparisons of gender differences in anticipatory and consummatory pleasure in OCD patients.

|  | **Male (n=71)** | **Female (n=59)** | ***t*** | ***P*** |
| --- | --- | --- | --- | --- |
| TEPS-ANT | 32.57±0.88 | 33.58±0.81 | -0.83 | 0.408 |
| TEPS-CONS | 36.15±1.08 | 38.57±1.13 | -1.55 | 0.124 |

Note: OCD, obsessive-compulsive disorder; TEPS: Temporal Experience of Pleasure Scale; TEPS-ANT:

anticipatory subscale of the TEPS; TEPS- CONS: consummatory subscale of the TEPS.

**Table S2**. Comparison anhedonia between OCD patients being treated and not being treated with medication

|  | **OCD patients with medication (n=57)** | **OCD patients without medication (n=73)** | ***t*** | ***P*** |
| --- | --- | --- | --- | --- |
| TEPS-ANT | 33.56±7.51 | 32.61±6.36 | -0.773 | 0.441 |
| TEPS-CONS | 37.25±9.37 | 37.26±8.64 | 0.009 | 0.993 |

Note: OCD, obsessive-compulsive disorder; TEPS: Temporal Experience of Pleasure Scale; TEPS-ANT:

anticipatory subscale of the TEPS; TEPS- CONS: consummatory subscale of the TEPS.

***Analysis of effect of insight on anhedonia in OCD***

To explore the effect of insight level on anhedonia in OCD, we did additional analysis. Firstly, the OCD patients were subdivided into patients with good insight (score of item 11 of YBOCS < 3, n = 102, OCD-GI) and patients with poor insight (score of item 11 of YBOCS ≥ 3, n = 28, OCD-PI) subgroups based on the insight level as evaluated by the insight item (item 11) of Y-BOCS. Secondly, the ANCOVA was used to compare the difference of anhedonia between OCD-GI and OCD-PI with the demographical and clinical variables demonstrating group differences controlled as covariates.

Results showed that OCD-GI and OCD-PI were similar in terms of age, education level, gender distribution, illness duration and depression level (*ps* > 0.05). OCD-PI had more sever symptom presentation than OCD-GI (*p* = 0.001). After controlling illness severity, OCD-GI and OCD-PI didn’t differ in either anticipatory or consummatory pleasure (*ps* > 0.05) (**Table S3** and **Table S4**). These results indicate that our results of anhedonia may be not impact by the insight level in OCD patients.

**Table S3** Comparisons of demographic and clinical characteristics between OCD patients with good and poor insight.

|  | **OCD-GI**  **(n=102)** | **OCD-PI**  **(n=28)** | ***t/χ^2^*** | ***p*** |
| --- | --- | --- | --- | --- |
| Age (years) | 23.61±9.06 | 23.46±6.81 | 0.078 | 0.938 |
| Education (years) | 12.93±3.14 | 13.20±3.81 | -0.338 | 0.707 |
| Gender (male/female) | 57/45 | 14/14 | 0.307 | 0.670 |
| BDI scores | 17.47±11.09 | 20.82±11.06 | -1.417 | 0.159 |
| Y-BOCS scores | 29.14±5.89 | 33.22±5.08 | -3.336 | 0.001 |
| Illness duration (years) | 3.58±4.96 | 4.95±4.11 | -1.341 | 0.182 |

Note: OCD, obsessive-compulsive disorder; OCD-GI, OCD patients with good insight; OCD-PI, OCD patients with poor insight; BDI, Beck Depression Inventory; Y-BOCS, Yale-Brown Obsessive-Compulsive Scale.

**Table S4** Comparisons of anticipatory and consummatory pleasure between OCD patients with good and poor insight.

|  | **OCD-GI**  **(n=102)** | **OCD-PI**  **(n=28)** | ***F*** | ***p*** |
| --- | --- | --- | --- | --- |
| TEPS-ANT | 33.18±5.97 | 32.48±9.61 | 0.328 | 0.568 |
| TEPS-CONS | 37.35±8.30 | 36.89±11.10 | 0.007 | 0.933 |

Note: OCD, obsessive-compulsive disorder; OCD-GI, OCD patients with good insight; OCD-PI, OCD patients with poor insight.
